# Perception of facial expressions reveals selective affect-biased attention in humans and orangutans

**DOI:** 10.1038/s41598-017-07563-4

**Published:** 2017-08-10

**Authors:** Carla Pritsch, Silke Telkemeyer, Cordelia Mühlenbeck, Katja Liebal

**Affiliations:** 1 Department of Education and Psychology, Comparative Developmental Psychology, Freie Universität Berlin, Berlin, Germany; 2 Research Group Diversity & Inclusion, Cognitive Sciences, Universität Potsdam, Potsdam, Germany; 3 Department of Psychology, Psychological methodology, Medical University Brandenburg Theodor Fontane, Neuruppin, Germany; 4 Department of Education and Psychology, Comparative Developmental Psychology, Freie Universität Berlin, Berlin, Germany

## Abstract

Rapid detection and recognition of another individual’s emotional state plays a pivotal role for humans and, most likely, other social species. Proper reactions contribute to an individual’s survival in potentially dangerous situations; this is ensured by a preferential attention towards salient cues. The predisposition to attend to certain categories of affectively salient stimuli– also referred to as affect-biased attention - is likely shared with other species, since fast detection of and appropriate reaction to threats is crucial to survival. We compared human children and one of our close relatives, Sumatran orangutans (Pongo abelii), and predicted that both look more attentively and longer at emotionally salient facial expressions of their own and corresponding other species, compared to neutral faces. However, in contrast to a bias towards emotions providing relevant information by indicating a threat, both species preferentially looked at the fear-related, but not the angry faces of humans and consistently preferred the silent-bared teeth espressions in orangutans. The differential attention towards certain expressions might derive from their social function and the need to detect a potential threat in the environment. Our findings are consistent with claims rooting this affect-biased attention characteristic of human perception in our evolutionary history.

## Introduction

In humans, physically salient stimuli selectively attract attention depending on the properties of the stimulus. Besides obvious physical properties salient stimuli may possess, like color or volume, emotionally valenced information also captures the individual’s attention more effectively in comparison to neutral stimuli (e.g., ref. 1). Salience, according to Todd *et al*.^2^ is defined as “the quality by which an aspect of the environment stands out relative to its surroundings” (p. 365). This predisposition to preferably attend to affectively salient stimuli over less salient, “neutral” stimuli is referred to as “affect-biased attention”^2^ and is shown in humans from an early age^3^.

In humans, facial and bodily expressions of emotions are a particularly salient source for triggering selective attention. Many studies found an attentional bias for affectively salient stimuli, such as preferential perception and enhanced memory when attentional resources are limited^4, 5^ and a greater likelihood to attract attention when viewing complex scenes^6^. Furthermore, there seems to be yet another layer of saliency within emotional cues attracting attention at different strengths. Valence could be shown to be a distinction criterion as many studies demonstrated that negative stimuli are even more salient, potent, and more efficacious than positive events^7^. Hence, it was argued that specifically negative emotional information has greater impact compared to positive information^8^. There exists evidence for a stronger impact of negative compared to positive information, in both basic and cognitively more complex psychological processes. For example, in conditional learning tasks, negative reinforcement results in faster and more permanent learning in humans and other animals^9^. In situations of judgment or decision-making, humans consider the negative aspects of a stimulus more than its positive aspects^10^. During impression formation, negative information, like immoral behaviors or bad traits, contributes more to the overall impression than positive information, even if both are of equal intensity^11, 12^. Humans also fixate negative stimuli longer, appraise them as being more complex and develop higher-level attributions to negative as opposed to positive stimuli^13, 14^, indicating that the processing of negative stimuli requires a superior amount of attention and more thorough processing^10^. The differential processing of negative and positive information is supported by a variety of neuroscientific studies^15^. Ito and colleagues measured event-related potentials in humans in response to pictures of negative and positive valence. They found that negative pictures elicited a significantly larger response than positive pictures, although the stimuli were controlled for probability, extremity and arousal^16^.

From an evolutionary perspective, the preferential perception of affectively salient information seems highly adaptive, since this information may signal potential threats in the environment. This selective attention process supports the fast and adequate reaction to such information, thus enabling the individual perceiving such information to avert injury or death^17^. Given its relevance for the individual’s survival, it seems very unlikely that the predisposition to attend to affectively salient stimuli is limited to humans. However, although there are some studies that have more generally investigated other animals’ (e.g. rodents and monkeys) approach or avoidance responses to positive and negative situations^9, 18, 19^, there is very little research explicitly examining whether species other than humans show a selective, affect-biased attention. For example, rhesus monkeys did not look differentially at facial expressions of conspecifics used in either aggressive or affiliative contexts^20^, while chimpanzees looked longest at videos showing agonistic interactions of conspecifics compared to videos with neutral or playful contents^21^. In a more recent study, bonobos were presented with a dot-probe paradigm^22^. This test measures the reaction time to detect a probe, which is presented on a screen at the position of a formerly presented neutral or emotional stimulus. The reaction time measured reflects the difficulty of disengaging attention from the former stimulus. The study demonstrated an affect-biased attention capture in this species, with greater attention capture of emotional in contrast to neutral scenes. Interestingly, the bonobos directed most attention to conspecifics’ positive emotional interactions and less to negative interactions.

Thus, the few existing studies on nonhuman primates report different findings with regard to the type of affective bias. Furthermore, no study yet exists which systematically compares humans and non-human primates in order to investigate whether the selective attention to negative information exhibited by humans is shared with other species, thus representing an evolutionary older trait. Even for humans, despite the already existing body of research, studies addressing the differences in selective attention towards negative versus positive emotional information have yielded controversial results^23–27^.

Therefore, to investigate the evolutionary roots of affect-biased attention, and specifically the role of negative emotional information, we compared human children at the age of 6–7 years with one species of great apes, Sumatra orangutans (*Pongo abelii*). We focused on children, because many studies show that children show an affect-biased attention with a tendency to put greater weight on negative cues^28, 29^, but have not yet developed a bias towards positive emotions, which is characteristic of older adults^24^. We used an eye-tracking paradigm to measure fixations at pairs of simultaneously presented human facial expressions of different emotions (fear, anger, happiness and neutral; Study 1) and four orangutan expressions. Since it is difficult to assign specific emotional states to facial expressions of nonhuman primates, we focused on those facial expressions, which are likely to occur in orangutans in similar contexts as in humans (silent-bared teeth display in insecure, potentially fearful situations; bulging lip display in agonistic interactions or in case of high tension; relaxed open mouth face in playful situations; and neutral expression; Study 2). We used two facial expressions of negative emotions with comparable characteristics, i.e. high arousal and threat-related, but which occur in two different contexts. While the expressions of fear might signal a threat in the environment motivating cooperative behavior such as informing peers, the expression of anger signals a direct threat from the sender to the perceiver^23, 30, 31^. If the affect-biased attention is selective with respect to semantically more salient negative information, being indicative of a potential threat, and shared by human and nonhuman primates, both species should preferentially look at facial expressions of negative emotions (anger, fear) compared to positive (happiness) and neutral faces and reveal comparable reactions in response to orangutan expressions.

## Results

**Figure 1:**
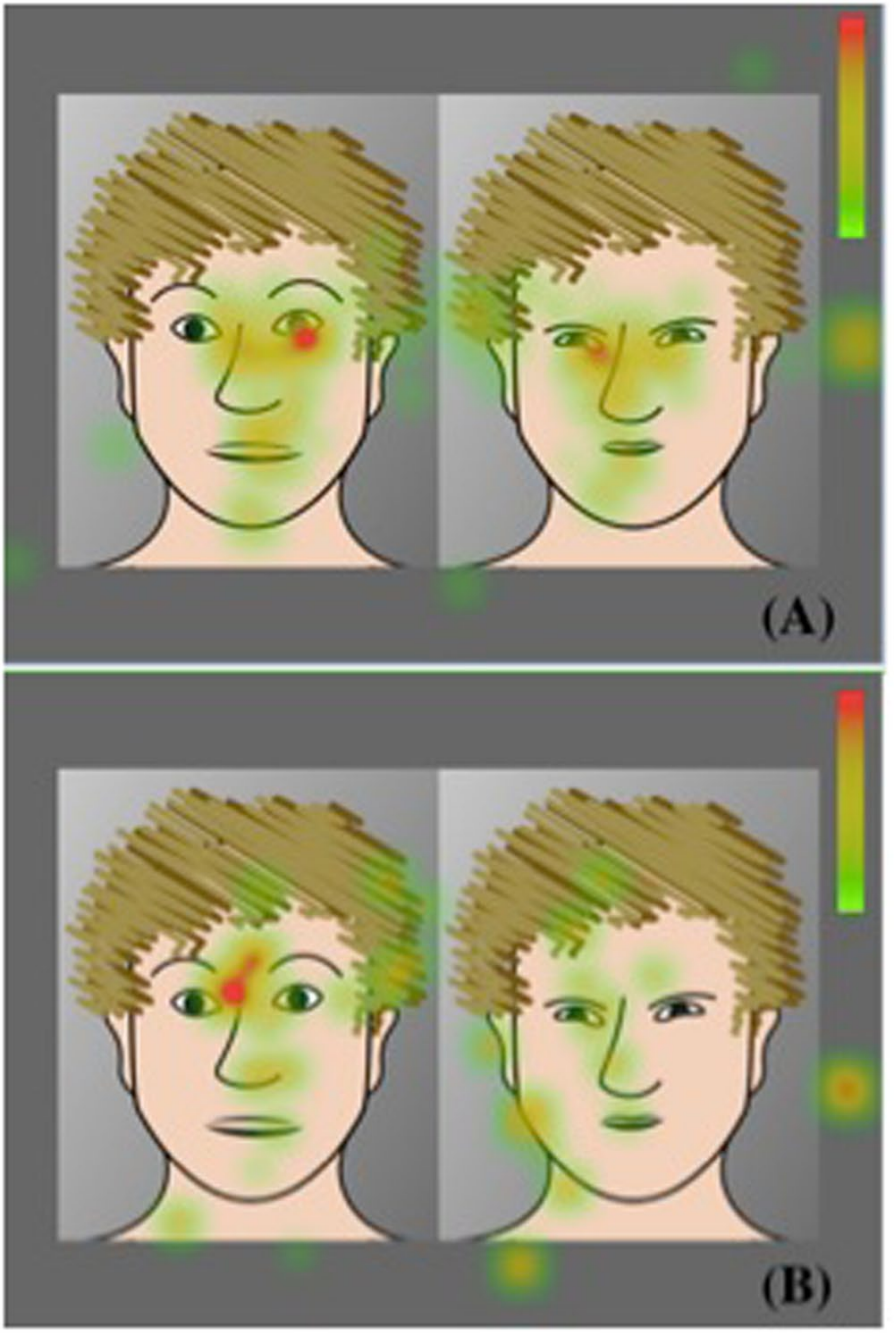
Visualizations (heat maps) of fixation durations. (**A**,**B**) Humans (**A**) and orangutans (**B**) fixate human facial expressions (fear vs. anger). While the color red marks those areas that are fixated longest, green indicates those areas that are fixated least. Important: because of copyright issues and publishing guidelines of the FACES database^63^, stimuli depicting human facial expressions are shown here only as schematic drawings, while in the study, we used the original photographs.

To compare gazing patterns across types of facial expressions, we measured *fixation rate* and *fixation duration* on two simultaneously presented different facial expressions (Fig. 1). We defined one of them as *target* and the corresponding other emotion as *distractor*. Each facial expression (fear, anger, happiness, or neutral) could either be *target* or *distractor*. We conducted two sets of analyses. First, we analyzed gazing patterns at the target emotion compared to *all* remaining other emotions (*all distractors*). Second, we analyzed gazing patterns at the target emotion combined with another *specific* emotion (*specific distractor*).

### Study 1: Gaze patterns at human facial expressions

**Figure 2:**
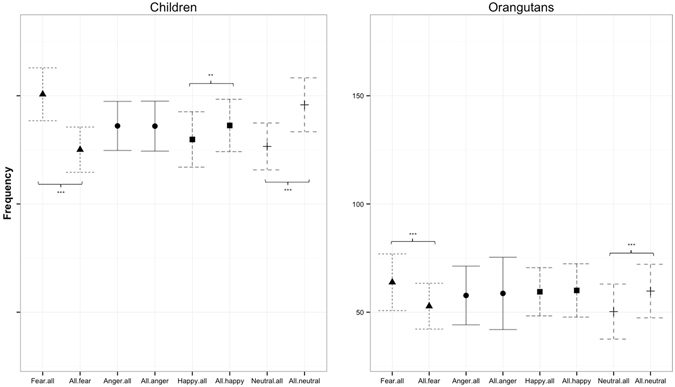
Fixation rates at human facial expressions: Mean estimates of the fixation rates at the target emotion (either Fear, Anger, Happy or Neutral) compared to all corresponding distractors (e.g. “Fear.all” = fear compared to happy, anger, neutral) for human children and orangutans. The corresponding combinations (e.g., Fear.all and All.fear) are depicted by the same symbols and lines. Significant comparisons are indicated by asterisks (>0.01*; >0.05**; >0.01***). Note that scales are identical for both plots.

Overall, orangutans fixated human facial expressions less frequently (rate: M = 231.29, SD = 7.2) and for shorter periods of time (duration: M = 76.92 s, SD = 2.95) than humans (rate: M = 543.13, SD = 10.85, duration: M = 193.94 s, SD = 3.67; Table S9). We therefore analyzed both species separately. First, the comparison of the target emotion with all remaining distractor emotions showed that both humans and orangutans fixated fearful human faces significantly more frequently (Fig. 2) and longer (Fig. S1) than the remaining distractor emotions (rate: humans χ^2^ = 34.31, df = 1, *p* < 0.001; orangutans χ^2^ = 8.58, df = 1, *p* = 0.003; duration: humans χ^2^ = 39.77, df = 1, *p* < 0.001; orangutans χ^2^ = 7.51, df = 1, *p* = 0.006). Furthermore, both species fixated neutral human faces less frequently and for less time than all other distractors (rate: humans χ^2^ = 19.95, df = 1, *p* < 0.001; orangutans χ^2^ = 10.03, df = 1, *p* = 0.002; duration: humans, χ^2^ = 13.37, df = 1, *p* < 0.001; orangutans χ^2^ = 3.82, df = 1, *p* = 0.051). Unlike orangutans, humans tended to fixate happy human faces more shortly and also less frequently than the rest of all other distractors (rate: χ^2^ = 5.49, df = 1, *p* = 0.019; duration: χ^2^ = 3.29, df = 1, *p* = 0.069) (see Tables S1 and S2 for the corresponding estimates, standard errors, confidence intervals and the t-values).

**Figure 3:**
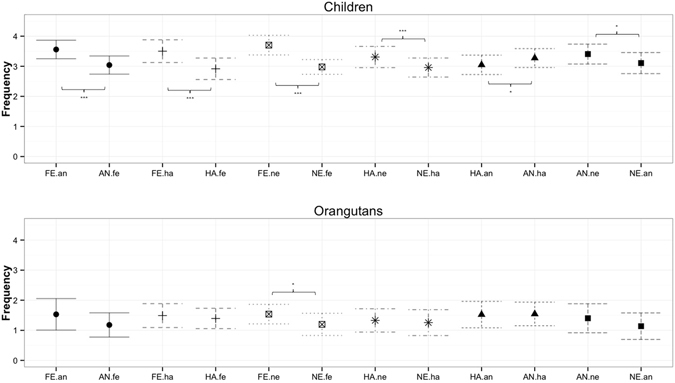
Fixation rates at specific pairs of human facial expressions. Mean estimates for the fixation rate for each target emotion (in capital letters, e.g. “FE”) compared to a specific distractor emotion (lower case letters, e.g., “an”) for human children and orangutans. The corresponding combinations (e.g., FE.an and AN.fe) are depicted by the same symbols and lines. fe = fear, an = anger, ha = happy, ne = neutral. Significant comparisons are indicated by asterisks (>0.01*; >0.05**; >0.01***).

Second, when comparing the target emotion with a specific distractor emotion, humans fixated fearful faces more often (Fig. 3) and longer (Fig. S2) than angry as well as happy or neutral faces, and showed a trend to fixate angry faces more than happy faces (rate: fear vs. anger: χ^2^ = 13.69, df = 1, *p* < 0.001; fear vs. happy χ^2^ = 15.92, df = 1, *p* < 0.001; fear vs. neutral: χ^2^ = 20.01, df = 1, *p* < 0.001, anger vs. happy χ^2^ = 3.47, df = 1, *p* = 0.063; duration: fear vs. anger: χ^2^ = 12.09, df = 1, *p* = 0.001; fear vs. happy χ^2^ = 14.46, df = 1, *p* < 0.001; fear vs. neutral χ^2^ = 16.78, df = 1, *p* < 0.001). Unlike orangutans, humans also fixated emotional faces more often and longer compared to neutral faces, with some of these findings being only marginally insignificant (rate: anger vs. neutral χ^2^ = 3.73, df = 1, *p* = 0.053; happy vs. neutral χ^2^ = 7.15, df = 1, *p* = 0.007; duration: anger vs. neutral χ^2^ = 3.60, df = 1, *p* = 0.058; happy vs. neutral χ^2^ = 3.36, df = 1, *p* = 0.067) (see Tables S3 and S4). For humans, our findings show an attentional bias that is modulated by the affective value of the stimuli, with fearful expressions being fixated most and longest. Like humans, orangutans also fixated fearful faces longer (but not more often) than angry faces (χ^2^ = 4.93, df = 1, *p* = 0.026) and showed a trend to fixate them more frequently than neutral faces (fear vs. neutral: χ^2^ = 2.91, df = 1, *p* = 0.088), but none of the other comparisons revealed significant differences. Together these results indicate that both species preferentially look at fearful facial expressions compared to angry and neutral faces, but unlike orangutans, humans also fixated angry facial expressions more and longer than happy faces, thus revealing an attentional bias towards affective stimuli expressing negative valence. Our findings for the perception of human facial expressions point to a particular attentional bias for the expression of fear, since humans as well as orangutans responded faster to and looked longer at fearful expressions.

However, the interpretations of the above reported findings in orangutans are certainly limited, because they looked at facial expressions of another species. Therefore, in a second study, we presented orangutan facial expressions to both species to investigate orangutans’ gazing patterns when looking at facial expressions of conspecifics, and whether humans show similar results when looking at facial expressions of a closely related species.

### Study 2: Gaze patterns at orangutan facial expressions

While research into human facial expressions usually focuses on the sender, inspired by Darwin’s (1872) approach to interpret them as expressions of felt emotions, facial expressions of nonhuman primates are commonly studied as displays, with focus on their adaptive function and their influence on the receiver^32^. Furthermore, it is problematic to assign certain emotional states to the facial expressions of other primates^33^. Comparative research on facial expressions across primates points to the difficulty that some facial expressions are present in a variety of species^34^, but that their use and function might differ across species^35, 36^. Therefore, this study represents a very first attempt to grapple with these questions by using facial expressions that occur in both orangutans and humans.

We chose to select orangutan facial expressions that have been observed in comparative contexts as our human stimuli pertaining to arousal and valence. The relaxed open mouth face is used in playful interactions, which form an emotionally positive context featuring high arousal, and appears in similar incarnations such as happy faces in humans^34^. The bulging lip face (or tense-mouth face) is described for many primate species^37^ and is used in situations involving tension or the motivation to attack; it has therefore been referred to as the ‘aggressive threat face’^37^ or an expression of anger^38^. The use of the silent-bared teeth face varies greatly between species as a function of their social structure and dominance relationships^35, 39^. In species with strict dominance hierarchies, it is usually used by subordinate individuals to communicate submission^40^, while in more egalitarian species it has a different function and is used as appeasement signal or to increase affiliative behaviors^34^. In orangutans, it occurs in situations when individuals are targets of others’ agonistic behaviors or as part of submissive behavior shown by subordinates towards approaching higher-ranking individuals^41^. Thus, although the function of this facial expression varies among species (van Hooff, 1967, Preuschoft 2004) and although little is known about facial communication of orangutans in general, our current knowledge indicates that this expression signals insecurity in socially tense situations (Liebal *et al*.^41^).

**Figure 4:**
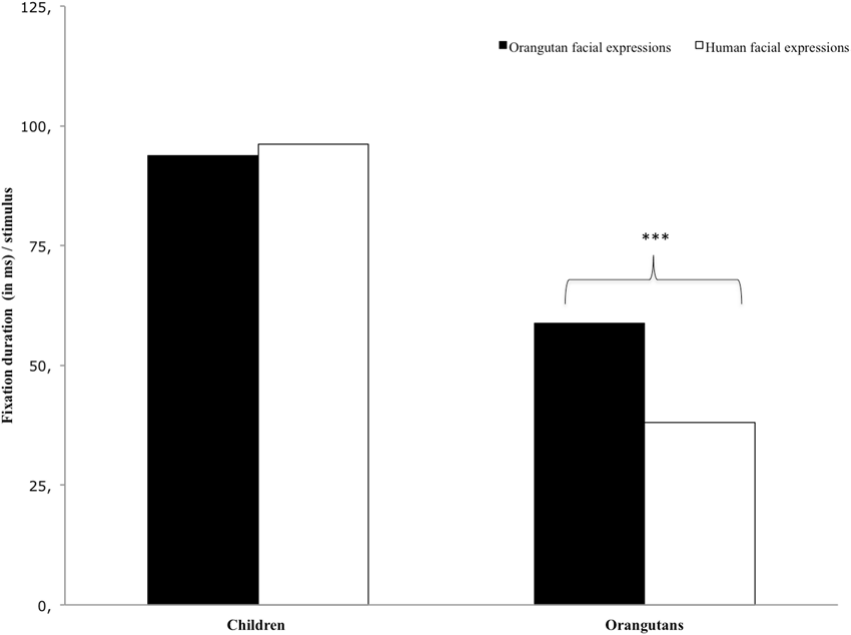
Fixation durations (in ms) per stimulus for children and orangutans on facial expressions of orangutans (black bars) and humans (white bars). Significant comparisons are indicated by asterisks (>0.01*; >0.05**; >0.01***).

Similar to Study 1, orangutans fixated facial expressions of conspecifics less often (rate: M = 444.31, SD = 4.54) and for shorter periods (duration: M = 135.60 s, SD = 1.54) than humans (rate: M = 631.18, SD = 6.05; duration: M = 216.02 s, SD = 2.59, Table S9). However, in comparison to Study 1, they fixated facial expressions of conspecifics significantly more often (fixation rate per orangutan stimulus: M = 0.19, SD = 0.025; fixation duration per orangutan stimulus: M = 58.85 s, SD = 8.39) than those of humans (fixation rate per human stimulus: M = 0.12, SD = 0.01; fixation duration per orangutan stimulus: M = 38.15, SD = 5.00; see Fig. 4 and Table S9).

**Figure 5:**
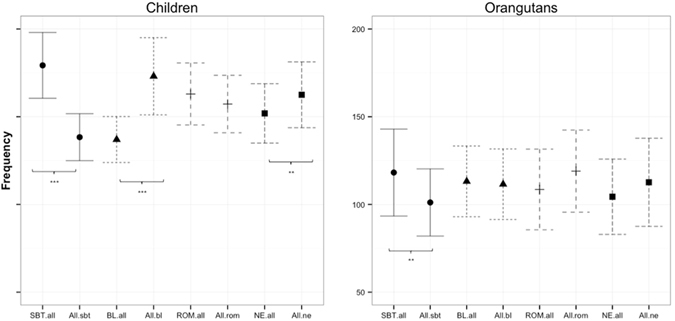
Fixation rates at orangutan facial expressions: Mean estimates of the fixation rates at the target emotion (either SBT (silent bared-teeth display), ROM (relaxed open mouth display), BL (bulging-lip display) and Neutral) compared to all corresponding distractors (e.g. “SBT.all” = SBT compared to ROM, BL and neutral) for human children and orangutans. The corresponding combinations (e.g., SBT.all and All.SBT) are depicted by the same symbols and lines. SBT: silent bared-teeth display; BL: bulging-lip display; ROM: relaxed open mouth display; NE: neutral. Significant comparisons are indicated by asterisks (>0.01*; >0.05**; >0.01***). Note that scales are identical for both plots.

First, when comparing the target emotion with all remaining distractor emotions, both orangutans and humans fixated the silent-bared-teeth face more frequently (Fig. 5) and longer (Fig. S3) than the corresponding distractor emotions, although this comparison was barely or marginally significant in orangutans (rate: humans χ^2^ = 21.25, df = 1, *p* < 0.001, orangutans χ^2^ = 3.91, df = 1, *p* = 0.048; duration: humans χ^2^ = 27.85, df = 1, *p* < 0.001, orangutans χ^2^ = 3.37, df = 1, *p* = 0.066). Unlike orangutans, humans fixated both the bulging lip face and the neutral face for shorter periods of time than their corresponding distractor emotions (rate: anger vs. all distractors of anger χ^2^ = 15.04, df = 1, *p* < 0.001, neutral vs. all distractors of neutral χ^2^ = 4.31, df = 1, *p* = 0.038; duration: anger vs. all distractors of anger χ^2^ = 17.14, df = 1, *p* < 0.001; neutral vs. all distractors of neutral χ^2^ = 7.08, df = 1, *p* = 0.008) (see Tables S5 and S6).

**Figure 6:**
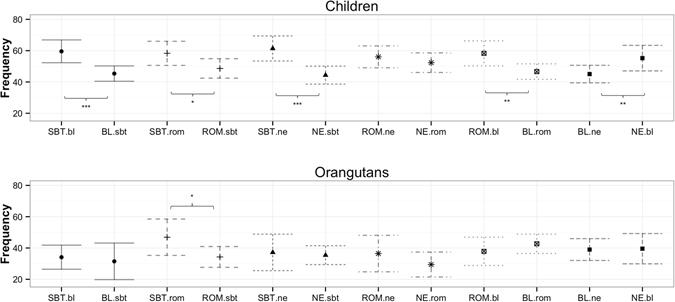
Fixation rates at specific pairs of orangutan facial expressions. Mean estimates for the fixation rate for each target emotion (in capital letters, e.g. “SBT”) compared to a specific distractor emotion (lower case letters, e.g., “ne”) for human children and orangutans. The corresponding combinations (e.g., SBT.ne and NE.sbt) are depicted by the same symbols and lines. SBT: silent bared-teeth display; ROM: relaxed open mouth display; BL: bulging-lip display, NE: neutral. Significant comparisons are indicated by asterisks (>0.01*; >0.05**; >0.01***).

Second, when comparing the target emotion with a specific distractor emotion, both humans and orangutans tended to fixate the silent-bared-teeth face more (Fig. 6) and longer (Fig. S4) than the relaxed open mouth face (rate: humans χ^2^ = 3.66, df = 1, *p* = 0.056, orangutans χ^2^ = 3.37, df = 1, *p* = 0.066; duration: humans χ^2^ = 5.01, df = 1, *p* = 0.025; orangutans χ^2^ = 3.56, df = 1, *p* = 0.059). Unlike orangutans, humans also fixated the silent-bared teeth face more frequently, but not longer, than neutral faces (rate: χ^2^ = 16.92, df = 1, *p* < 0.001). Humans also fixated the bulging lip face less often and for shorter intervals compared to each of the other facial expressions (rate: bulging lip face vs. neutral χ^2^ = 4.03, df = 1, *p* = 0.045; bulging lip face vs. relaxed open mouth face χ^2^ = 5.68, df = 1, *p* = 0.017; bulging lip face vs. silent bared teeth face χ^2^ = 9.23, df = 1, *p* = 0.002; duration: bulging lip face vs. neutral χ^2^ = 3.74, df = 1, *p* = 0.053; bulging lip face vs. relaxed open mouth face χ^2^ = 5.49, df = 1, *p* = 0.019; bulging lip face vs. silent bared teeth face χ^2^ = 13.57, df = 1, *p* < 0.001) (see Tables S7 and S8).

## Discussion

Our findings support the assumption of an attentional bias for affectively salient information in humans and orangutans. However, the hypothesis that human children and orangutans selectively attend to facial expressions of negative valence could only partially be confirmed. We found that both species specifically looked more and longer at fearful, but not angry facial expressions in humans (Study 1). Equally, both species looked longer at the silent bared-teeth face but not at the bulging lip face (Study 2). When looking at human facial expressions, human children also preferentially looked at angry faces compared to positive and neutral information. However, angry faces were still fixated for significantly shorter intervals and less often than fearful expressions. Furthermore, both species looked in general less at neutral compared to positive or negative faces.

Together these findings support our assumption that increased attention to emotional faces is an adaptive trait, since it is present in humans and orangutans and it is not limited to looking at emotional facial expressions of their respective own species. The finding that in humans fear is preferentially processed compared to anger can be explained by the fact that each of these two emotions and their corresponding expressions convey different information, which requires different responses and most importantly, is of different relevance for the individual^42^. Fearful expressions signal the detection of a potential threat in the environment, resulting in an increased state of alertness of the individual perceiving this signal and motivating further attention to localizing the potential threat^43^. Anger, on the other hand, seems to function as a socially regulatory emotion^44^. In humans, the expression of and reaction to anger is highly dependent on cultural display rules^45^, and both the expression as well as the appropriate response patterns become increasingly refined with progressing age and integration in social groups^46, 47^. Because of their different functions, it has been suggested that the perception of fearful and angry expressions follows different developmental trajectories. Infants at the age of 4–9 months can already differentiate several types of facial expressions including happiness, anger and fear^48, 49^. This ability is increasingly refined until late adolescence, but is delayed for some emotions, including anger^47, 50^. It could therefore be hypothesized that children in the current study did not yet exhibit a more mature response to anger and that the observed fear bias reflects the lack of a fully developed bias towards negative emotions including anger. An alternative explanation could be that children are least familiar with fearful facial expressions and thus react more strongly to the novel stimulus. Children mostly see neutral, happy and maybe angry faces, but are less likely to be confronted with fearful facial expressions. Several studies have confirmed that children from the second half of the first year pay more attention to negative emotions, more precisely fear. For instance, at seven months of age, infants look longer at negative (e.g., fearful) as opposed to positive (e.g., happy) facial expressions^51–53^. Event-related potential (ERP) studies reveal that seven-month-olds show greater amplitudes in the Negative-central (Nc) component in reaction to fearful as compared to happy faces^51, 54^, indicating that fearful facial expressions attract higher levels of attention than happy expressions in these young children. This greater attention to negative cues was attributed to an infant’s onset of independent exploration of the environment. During this time, infants begin to encounter potentially negative situations and experience the corresponding negative emotions (insecurity/fear), prior to having developed a refined and intensified understanding of negative emotions. Consequently, familiarity might be a potential driver to attend to certain stimuli more strongly, but the fact that human adults were shown to attend to fearful expressions more strongly as well rather hints at a bias based on the expressions as such^7^.

The additional finding that humans and orangutans looked longer at emotional in contrast to neutral facial expressions confirms the attentional capture of emotionally salient cues. The preferential fixation of the silent bared-teeth over other emotional expressions requires further investigation, since little is known about the social function of the silent bared-teeth display in orangutans yet. Therefore, we need to be cautious with regard to assigning a specific underlying emotional state to this facial expression.

The finding that both species show comparative preferences towards facial expressions of their own and the corresponding other species is somewhat surprising. While orangutans had extended experience of interactions with humans, which could at least partly explain this finding, the children had not previously interacted with orangutans. It could be argued that since facial expressions of humans and other primates may represent homologous structures^55^, the processing of facial expressions is similar across species. This seems at least partly supported by Kano^56^, who demonstrated that, despite the fact that humans and chimpanzees showed different, species-specific scanning patterns when looking at faces of humans or chimpanzees, these patterns where consistent within each species regardless of whether they looked at faces of their own or other species. However, it is important to note that despite similarities of form across species, the communicative function of facial expressions might be different across species, which is specifically true for the silent-bared teeth face^39^. Furthermore, our findings in Study 2 are still of a preliminary nature for the two following reasons. First, very little is known about facial expressions and their social use in orangutans^41, 57, 58^. This particularly concerns the use of the silent-bared teeth face, which serves different functions across species and contexts^32, 34–36^. Although Liebal and colleagues^41^ have observed it mostly in agonistic and submissive interactions used by subordinate individuals, its social function and how exactly it influences the recipient’s behavior have never been studied systematically in orangutans. Based on the fact that this expression is shown in potentially fearful or socially tense situations, we used it as a negative emotion in this study. However, the conclusions are yet preliminary and require additional testing as it remains unclear whether conspecifics perceive it as an expression of fear. Second, the stimuli depicting different orangutan facial expressions were not as standardized and extensively rated as the human stimuli. To obtain more resilient findings, the establishment of a comprehensive database of orangutan faces is needed, with pictures that capture their facial expressions from comparable angles, in very good quality, and controlling for several features such as background and brightness. Furthermore, we need stimuli from a larger number of individuals, each of them producing a variety of facial expressions to control for the identity as well as age and sex of the individual showing the facial expression. It is also worth mentioning that we did not systematically assess exclusion criteria for the participating children, such as the children’s mental state or visual abilities. However, the headmaster and the involved teachers recommended children on the basis of their suitability for our study. For the orangutans, the broad age range is a potential limitation. Obtaining a great number of orangutans for an eye-tracking study is challenging. However the aim for a next study could be an increase in the number of individuals.

Despite these limitations, our findings provide evidence for a selective attention bias based on the emotional value of the facial expression, which might be based on the salience of the transmitted information and, ultimately, the social significance for the individual that perceives this information. Thus, the fast and appropriate response to distinct facial expressions plays a crucial role in both humans and orangutans, and most likely represents a trait shared by all primates.

## Method

### Participants

#### Orangutans

We tested eight orangutans (3 males, 5 females) ranging from 4 to 33 years (M = 14.9, SD = 10.45) housed at the Wolfgang Köhler Primate Research Center (WKPRC) of the Max Planck Institute for Evolutionary Anthropology at Leipzig Zoo (Germany). They had previously participated in studies using eye-tracking techniques and therefore were familiar with attending information presented on a screen. They live in their social group in adjacent semi-natural indoor (230 m²) and outdoor (1680 m²) enclosures equipped with trees, ropes and regular enrichment activities. Testing was conducted in an indoor testing room (25 m²). For the purpose of this study, the apes were neither food- nor water-deprived and received their regular feedings, daily enrichment and water ad libitum. They participated voluntarily and were able to stop participating at any time.

#### Humans

For *Study 1* (human faces), we tested 24 school children from grades 1 and 2, ranging from 6–7 years of age (M = 6.7, SD = 0.45; 13 male/11 female). For *Study 2* (orangutan faces), we tested 20 additional school children from grades 1 and 2, ranging from 6–7 years of age (M = 6.7, SD = 0.48; 9 male/11 female). All children were recruited from the Grundschule Niederkaufungen, Hessen, Germany. None of them had previously participated in studies using eye-tracking techniques, but all of them had experience in observing pictures on a screen (TV, Computer).

#### Ethics statement

This study with orangutans was approved by an ethical committee consisting of researchers of the Max Planck Institute for Evolutionary Anthropology and zoo keepers (Prof. M. Tomasello, Dr. J. Call, Dr. D. Hanus, veterinarian Dr. A. Bernhard, head keeper F. Schellhardt and assistant head keeper M. Lohse). This research was conducted in accordance with the recommendations of the Weatherall report “The Use of Non-human Primates in Research”^59^, the requirements of the European Association of Zoos and Aquaria^60^, the World Association of Zoos and Aquariums^61^ and the “Guidelines for the Treatment of Animals in Behavioral Research and Teaching” of the Association for the Study of Animal Behavior^62^. The WKPRC does not give permission to conduct any invasive research.

Research on the human sample was conducted according to the ethical standards of the Deutsche Gesellschaft für Psychologie (DGPs; German Psychological Association) and the ethical guidelines of the research institution (Freie Universität Berlin) as approved by the department of Comparative Psychology under supervision of Prof. Dr. Katja Liebal and the graduate school of the cluster ‘Languages of Emotion’. Additionally, the headmaster and the involved teachers approved the study and informed the parents. We obtained informed consent from the parents prior to testing and oral consent from the children before they participated in the study. Hence, informed consent was obtained for all human subjects in accordance with the Declaration of Helsinki.

### Stimuli

#### Study 1 - Human facial expressions

Stimuli were obtained from the FACES Database, a set of photographs of humans from different age classes performing various facial expressions. Photographs were standardized in size, color and background, and have been extensively rated as regards their emotional content before being published^63^.

**Figure 7:**
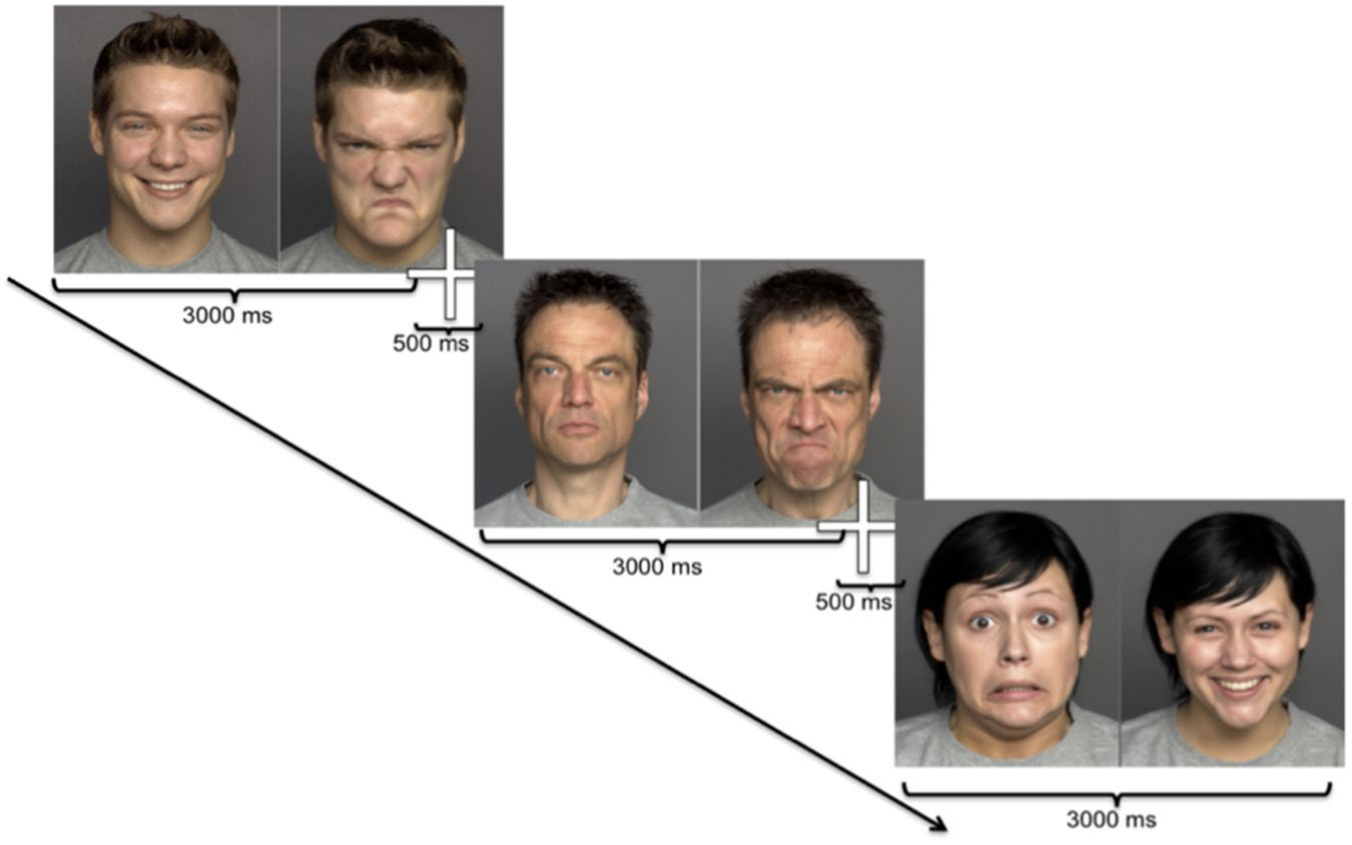
Temporal sequence of the presentation of human facial expressions. Each stimulus consisted of two portrayal pictures and was presented for 3000 ms, followed by a fixation stimulus (500 ms), before the next stimulus appeared. For human facial expressions, each stimulus (from FACES^63^), consisted of two pictures of the same individual showing two different out of four facial expressions (fear, anger, happiness, or neutral).

To create our stimuli, we selected a subset of 14 young Caucasian adults (seven males, seven females) performing happy, fearful, angry, and neutral faces. Each stimulus comprised two portrayal pictures, each with a different facial expression of the same person (Fig. 1). For each of the 14 individuals, we combined all possible combinations of the four types of facial expressions, without combining the same facial expression. The size of each portrayal picture was 440 × 550 pixels; the size of each stimulus consisting of two portrayal pictures was 890 × 550 pixels. Facial expressions were presented on grey background with a 10 pixel-gap between the two portraits. The presentation of each facial expression (left or right) was counterbalanced and the order of presentation of the stimuli was randomized. Each stimulus was presented for 3000 ms, separated by a central fixation stimulus (orangutans: grapes, bananas; humans: fixation cross) shown for 500 ms in order to attract the participants’ attention to the center of the screen before the next stimulus appeared (Fig. 7).

#### Study 2 - Orangutan facial expressions

**Figure 8:**
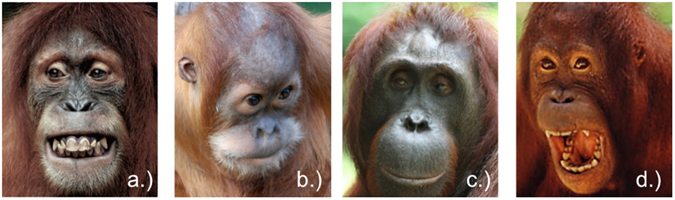
Orangutan stimuli. (**a**) Silent bared-teeth (SBT): AU10 (upper lip raiser) + AU12 (lip corner puller) + AU25 (lips part)^58^. AU combination of AU10+12+25 is described as silent-bared teeth display (SBT) for chimpanzees^55, 65^. (**b**) Bulging lip display (BL): Presence of AU24 (lip presser)^58^. Source: Prof. Dr. Katja Liebal. (**c**) Neutral face (NE): AU0^58^ Source: Prof. Dr. Katja Liebal. (**d**) Relaxed open mouth (ROM): AU10 (upper lip raiser) + AU12 (lip corner puller) + AU25 (open mouth) + AU27 (mouth stretch). The playface is described for chimpanzees as AU combination AU12+25+26^55, 65^ or AU12+25+27^65^. For gorillas the playface is described as AU16+25+26 and as „full playface“ within play context as AU10+16+25+26^68^. Source: Prof. Dr. Katja Liebal.

Since there is currently no database with standardized, frontal portraits of orangutan facial expressions available, we obtained footage from a collection of pictures from the WKPRC, Leipzig, and from the internet. We also took pictures of orangutans’ facial expressions in different zoos (Leipzig, Zürich). We selected pictures of facial expressions that orangutans show in comparable emotional situations as those underlying the human facial expressions: the silent-bared teeth display occurs in contexts of insecurity, submissive/affiliative behavior or fear^39, 64^; the bulging lip face is shown in contexts of frustration, similar to the angry facial expression in humans^65^; the relaxed open mouth face, which occurs in playful interactions^41^; and a neutral face. Thus, while we tried to select types of orangutan facial expressions that were similar to human facial expressions with regard to the type of emotion they expressed, they differed between species in at least some of their formal features (for examples, please see Fig. 8). Each type of facial expression was represented by five different individuals of different ages, but no adult flanged males were included to avoid an impact on the participants’ gazing patterns, because of their distinct head morphology. Each stimulus comprised two portrayal pictures, each with a different facial expression. However, since there is no extensive, standardized database for orangutan facial expressions, orangutan stimuli included two portrayals showing facial expressions of two *different* individuals and not the same individual as was the case for human stimuli. The size of each portrayal picture was 440 × 550 pixels; the size of each stimulus consisting of two portrayal pictures was 890 × 550 pixels. We paired all possible combinations of the four types of facial expressions, without combining the same facial expression. Facial expressions were presented with a 10 pixel-gap between the two portraits. The presentation of each facial expression on the left or right side of the screen was counterbalanced and the order of presentation of the stimuli was randomized. Each stimulus was presented for 3000 ms, separated by a central fixation stimulus (orangutans: grapes, bananas; humans: fixation cross) shown for 500 ms in order to attract the participants’ attention to the center of the screen before the next stimulus appeared.

All stimuli were presented to both humans and orangutans. The only difference in the procedure between species was that orangutans were presented with various subsets of the stimuli in order to account for their shorter attention span. For human stimuli, orangutans received 14 sessions on separate days, with one session comprising 12 experimental stimuli (3000 ms) and 12 fixation stimuli (500 ms), resulting in a total duration of 42 seconds per session. Humans received only one session comprising a total of 168 different stimuli, resulting in a total duration of 9.8 minutes. For orangutan stimuli, orangutans were tested in 16 sessions on separate days, with one session comprising 12 experimental stimuli (3000 ms) and 12 fixation stimuli (500 ms), resulting in a total duration of 42 seconds per session. Humans received only one session comprising a total of 192 different stimuli, resulting in a total duration of 11.2 minutes.

### Apparatus

The eye-tracking apparatus was used in both studies and is a table-mounted, 17-inch, monitor-integrated (1024 × 768 pixels) Tobii eye tracker T60 (60 Hz, Tobii Technology AB, Stockholm. Sweden). It operates on a corneal reflection-based system and measures participants’ gaze movements and fixations. The eye-tracker records both eyes simultaneously and employs wide-angle lenses that allow for a relatively large range of head movements without head-restraining devices. We used the same setting for both species with only very few adjustments to the corresponding testing conditions.

### Procedure

#### Orangutans

The orangutans voluntarily entered the testing room from the indoor enclosure and were able to freely move in an experimental booth, separated from the experimenter and the eye-tracking apparatus by a transparent acrylic panel. Before the test started, they were enticed into position in front of this panel using grape juice, which they could sip out of a tube attached to the panel. First, orangutans’ eyes were calibrated using a Tobii Studio (2.1) automated three-point calibration tool, which uses three red points pausing in extreme positions (in two corners) and in the center against a green background. This calibration was necessary to adapt the eye-tracker to the individual characteristics of their eyes and to create the corresponding eye model. Calibration was repeated until maximum accuracy was achieved. After successful calibration, the subjects received one session with one subset of stimuli per day. During testing, orangutans received grape juice to position them in front of the screen and to keep their attention, according to the technique established by Kano and colleagues^66^. After each session, they were rewarded with some fruit pieces regardless of their performance. If they poorly attended to some of the subtests with lost gaze data for more than 50% of the stimuli, we repeated those sessions and later used repeated measurements to analyze the data to account for repeated testing.

#### Humans

Humans were tested in a separate room in their school and were instructed to explore the stimuli on the screen. Before testing, all participants gave their informed consent. We used the Tobii Studio (2.1) automated calibration tool for humans consisting of five red points moving on a green screen and pausing in five random positions (calibration points, four corners and the center). The participants were asked to follow and fixate the red dot. Calibration was repeated until the maximum accuracy was obtained, which was followed by one test session. This procedure was identical for both studies.

### Data Analysis

We focused on the two dependent variables *fixation duration* and *fixation rate*. We defined each picture of a facial expression as an area of interest (AoI). Consequently, each stimulus comprised two separate AoIs (each of the two portraits with 440 × 550 pixels in rectangular shape). Fixations were only measured if they were executed within one of these two AoIs. We scored a fixation if the gaze remained stationary for at least 100 ms within a radius of 50 pixels (more than five measurement samples at a 60 Hz sampling rate; Tobii fixation filter).

We used the software R^67^ to analyze the data. We ran separate models for orangutans and humans due the large difference in their general attention to the stimuli. Thus, we analyzed differences within each species and discussed these patterns between both species. Since each stimulus consisted of two emotional facial expressions representing the *emotion combination*, we defined a specific *target*, representing one of the presented emotions, and a specific *distractor* (a specific emotion presented together with the target = specific distractor) or a specific set of distractors (all emotions being presented with the target on one stimulus = all distractors). Depending on the focus of the analysis, each emotion (fear, anger, happiness, or neutral) could either be a *target* or *distractor*. We ran a linear mixed model with no intercept (and no slopes), because all predictors in our model were dummy-coded levels of the predictor variable *emotion combination*. The final model thus comprised the value of the respective dependent variables, *fixation rate* and *fixation duration*, as a response, the different levels of the predictor *emotion combination* (dummy coded without a reference category) as fixed effects, and *subject* as random effect to account for individual differences between subjects of a group. The response was represented by the value of the respective dependent variables *fixation rate* and *fixation duration*. Since the different levels of the predictor variable *emotion combination* are not independent, we allowed for all possible correlations in our model.

We visually inspected the qq-plot and the residuals plotted against fitted values to test whether the assumptions of normal distribution and homogeneity of residuals were met, but found no violation of these assumptions. P-values of the different effects, i.e. differences between the simultaneously presented facial expressions (*target emotion vs. distractor*), were derived by comparing the full model with all levels of *emotion combination* included and a (special) reduced model; this model comprised all factors present in the full model (dummy coded factor levels of *emotion combination*), but with the comparison of the target and its respective distractor(s), that is the comparison in focus, set to equal (e.g. I(*Fear vs. Anger*)). This procedure enabled us to contrast the fit of the full model against a reduced model disregarding a specific comparison. If this full-reduced-model comparison was significant, we concluded that the model fit without the specific comparison is significantly worse and, thus, the comparison itself is of significant difference. The significance of this comparison (full model against reduced model) was tested using a likelihood ratio test (R function anova with argument test set to “Chisq”).

Two different sets of analyses were conducted in order to compare a target to either an entire set of emotions (*all distractors*) or to a specific target emotion (*specific distractor*). The multilevel analyses were similar for both comparisons except for the different factor levels, representing either the comparison of one emotion to all distractors or to a specific distractor.

#### Target emotion vs. all distractors

The aim was to investigate whether a specific emotion has an effect on the gazing patterns regardless of its combination with a specific *distractor* emotion. Therefore, we compared the fixation duration as well as the fixation rate for each target emotion to the average fixation duration and fixation rate of all corresponding remaining *distractors*, which were all other emotions except the target emotion (for example, target = *Fear vs*. all distractors = *Anger* + *Happy* + *Neutral*). The estimates of this analysis described one level of the dummy-coded factor *emotion combination*, reflecting one target emotion and all corresponding distractors shown with the target emotion on the same stimulus. Since we did not integrate an intercept (or slopes), the estimates in the corresponding plots (Figs 2 and 4) represent single values of the fixation duration or the fixation rate. A linear mixed model calculated the estimated values for the presented target emotion in contrast to its corresponding distractors (see above).

#### Target emotion vs. specific distractor

We examined the gazing pattern at the *target* emotion depending on its combination with another *specific distractor*. Therefore, we compared the fixation duration as well as fixation rate of each target emotion to another specific distractor emotion (e.g. target = *Fear vs*. specific distractor = *Anger*).

The corresponding analyses for the fixation durations and fixation rates were similar to those described above, except of the factor levels representing each specific emotion combination, which constitute one level of the dummy-coded factor *emotion combination*, consisting of the target emotion and a specific distractor emotion (e.g. *Fear vs. Happy*). Intercepts or slopes were not included in the model, because there was no reference category implemented in this analysis. Consequently, estimates are represented as single values (estimates).

## Electronic supplementary material

Supplementary Material
