# Supplementary material for: Perception of facial expressions reveals selective affect-biased attention in humans and orangutans
Source: Sci Rep. 2017 Aug 10;7:7782. doi: 10.1038/s41598-017-07563-4 (PMC5552869; doi:10.1038/s41598-017-07563-4)
Supplement: Supplementary file 1 — Supplementary Material [file 41598_2017_7563_MOESM1_ESM.pdf]

Title: Perception of facial expressions reveals selective affect-biased attention in humans and orangutans

Authors: Carla Pritsch[1]*, Silke Telkemeyer[2], Cordelia Mühlenbeck[3], Katja Liebal[4]

Affiliations:

[1] Graduate School "Languages of Emotion", Department of Education and Psychology, Freie Universität Berlin, Berlin, Germany

[2] Research Group Diversity & Inclusion, Cognitive Sciences, Universität Potsdam, Germany

[3] Department of Psychology, Psychological methodology, Medical University Brandenburg Theodor Fontane, Neuruppin, Germany

[4] Department of Education and Psychology, Comparative Developmental Psychology, Freie Universität Berlin, Berlin, Germany

Correspondence to: carla.pritsch@fu-berlin.de

Supplementary Materials

Stimuli

Example orangutan silent-bared teeth vs. human fear expression: While the fearful expression in humans consists of raised brows, widely open eyes exposing a large amount of white sclera, a slight mouth opening showing no or only bottom teeth tips, in great apes, this facial expression is also referred to as the bared-teeth display characterized by retracted lip corners and a raised upper (and lower) lip exposing the teeth (*43*). To ensure that we included the correct pictures for each type of facial expression, Manuela Lembeck, a certified FACS coder for human facial expressions as well as for orangutans, chimpanzees and gibbons checked the structural features of each picture.

Data extraction

We exported the raw data using the software Matlab (R2014b). All participants were included into the analyses, but 9.0 % of all trails of orangutans and 3.6 % of all trails of humans had to be excluded since they did either not look at one of the predefined AoIs or did not attend to the stimulus for at least 500 ms (of the 3000 ms). In case of multiple measurements of the same stimulus due to replicated sessions in orangutans, we averaged the individual's performance for the replicated trials of the respective session.

Data Analysis with R

To test our hypothesis that gazing patterns differ as a function of valence of the emotions, we treated observations as repeated measures nested in subjects. Since our predictors (target emotion vs. distractor emotion) were categorical, we had to dummy code the respective combination of emotional facial expressions (*emotion combination*), with each combination representing one factor level and no reference category being implemented. We fitted a linear mixed model in R using the function lmer of the R-package lme4 (*48*).

Figures & Tables

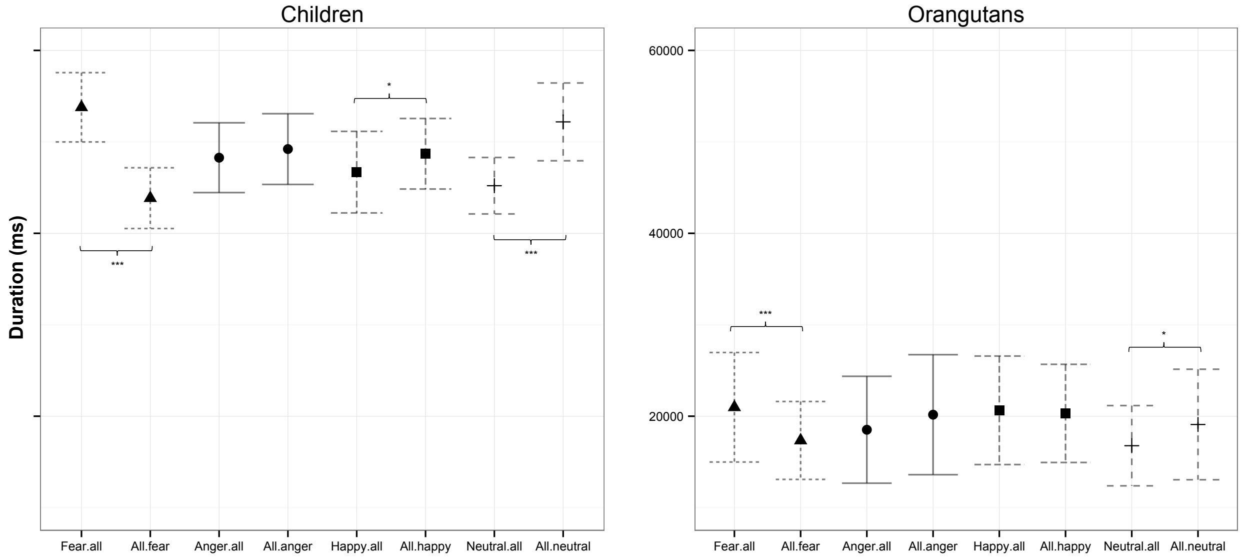

Fig. S1: Fixation duration at human facial expressions: Mean estimates of the fixation duration at the target emotion (either Fear, Anger, Happy or Neutral) compared to all corresponding distractors (e.g. "Fear.all" = fear compared to happy, anger, neutral) for human children and orangutans. The corresponding combinations (e.g., Fear. all and All.fear) are depicted by the same symbols and lines. The scales (in ms) are the same for both plots.

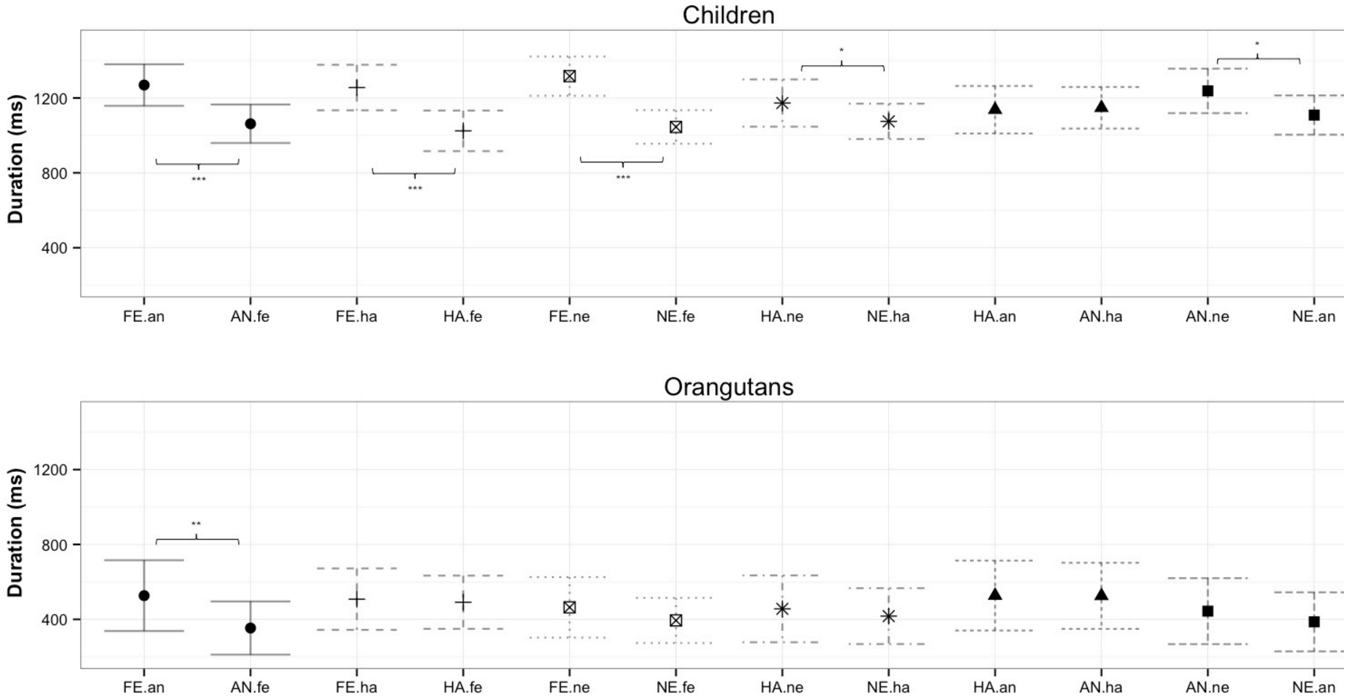

Fig. S2: Fixation duration (ms) at specific pairs of human facial expressions. Mean estimates for the fixation duration for each target emotion (in capital letters, e.g. "FE") compared to a specific distractor emotion (lower case letters, e.g., "an") for human children and orangutans. The corresponding combined pairs (e.g., FE.an and AN.fe) are depicted by the same symbols and lines. Fe:fear, an=anger, ha=happy, ne=neutral.

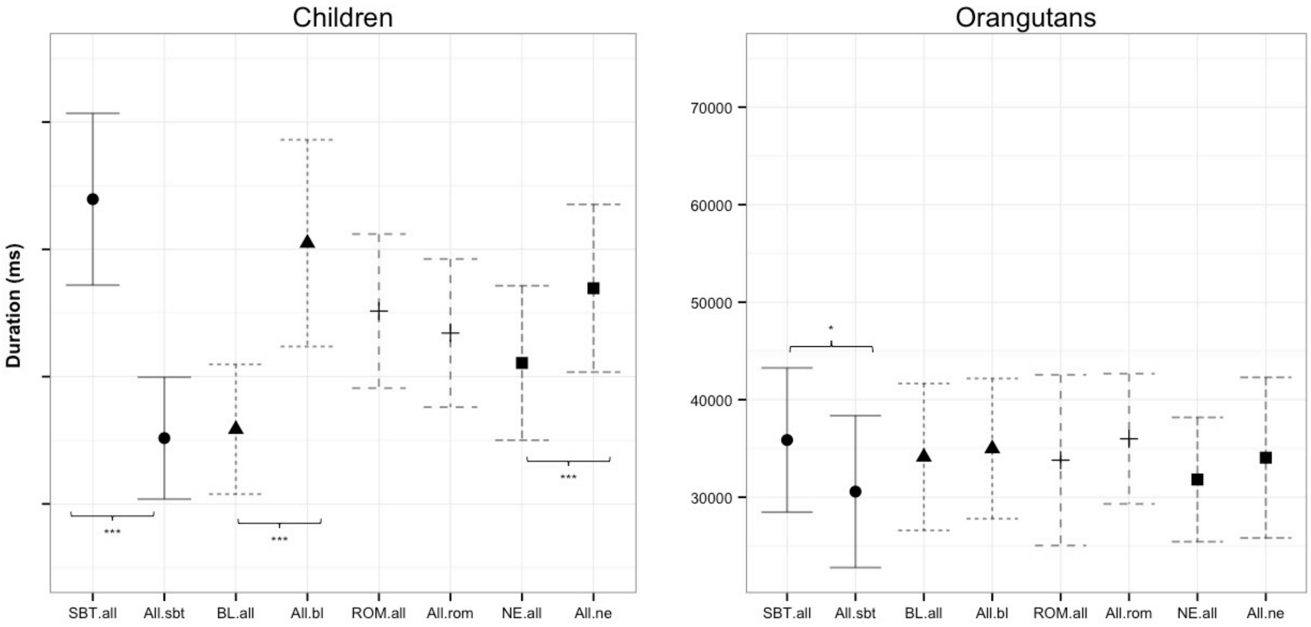

Fig. S3: Fixation duration (ms) at orangutan facial expressions: Mean estimates of the fixation duration at the target emotion (either SBT (silent bared-teeth display), ROM (relaxed open mouth display), BL (bulging-lip display) and Neutral) compared to all corresponding distractors (e.g. "SBT.all" = SBT compared to ROM, BL and neutral) for human children and orangutans. The corresponding combined pairs (e.g., SBT. all and All.SBT) are depicted by the same symbols and lines. The scales are the same for both plots. SBT: silent bared-teeth display; BL: bulging-lip display; ROM: relaxed open mouth display; NE: neutral.

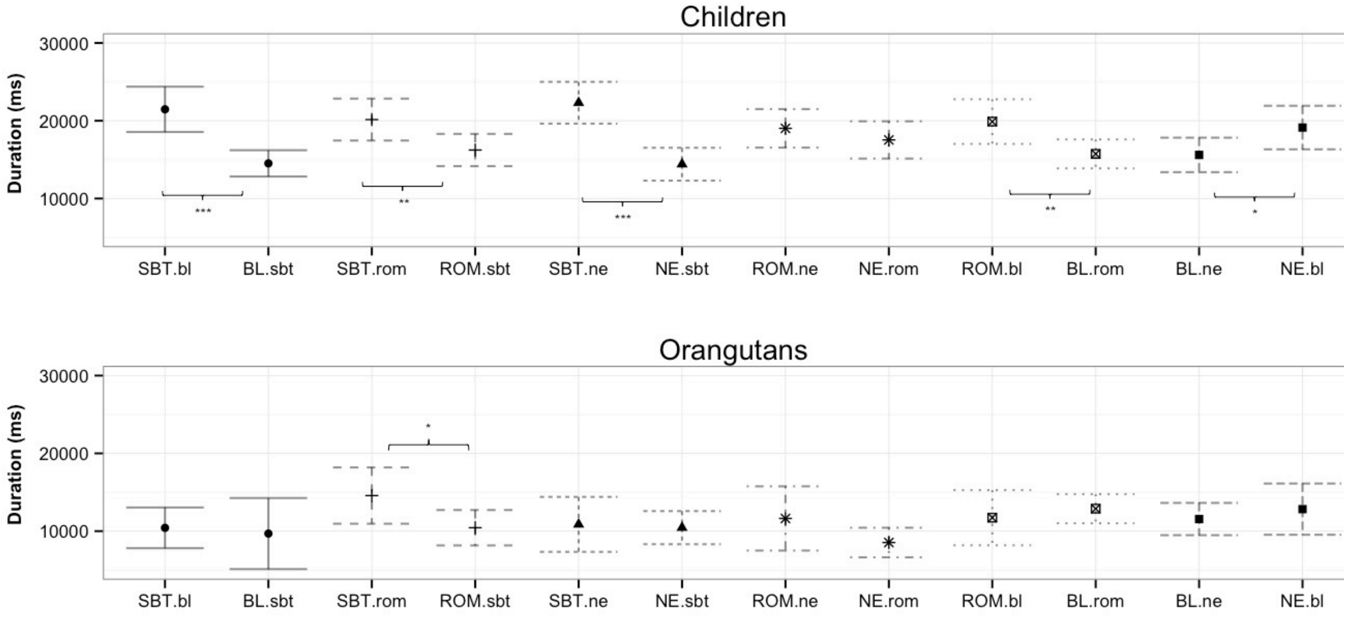

Fig. S4: Fixation duration (ms) at specific pairs of orangutan facial expressions. Mean estimates for the fixation duration for each target emotion (in capital letters, e.g. "SBT") compared to a specific distractor emotion (lower case letters, e.g., "ne") for human children and orangutans. The corresponding compared pairs (e.g., SBT.ne and NE.sbt) are depicted by the same symbols and lines. SBT: silent bared-teeth display; ROM: relaxed open mouth display; BL: bulging-lip display, NE: neutral.

Tables S1-S8

Table S1: Fixation rate estimates at the target emotion compared to all corresponding distractor emotions for human children (Hum) and orangutans (Ape) looking at human facial expressions. Values are derived from general mixed linear model (GLMM) using the function ‘summary’, predicting estimates, standard errors (Std. Error), upper and lower confidence intervals (upper CI, lower CI), and t-values for the mean fixation rate of Children (Hum) and orangutans (Ape) on a particular target emotion (fear, anger, happy, neutral) compared to the corresponding all other distractor emotions (All.fear=neutral, happy, anger; All.anger=neutral, happy, fear; All.happy=neutral, fear, anger; All.neutral=fear, anger, happy).

Emotion combination	Estimate		Std. Error		Upper CI		Lower CI		t-value	
	Hum	Ape	Hum	Ape	Hum	Ape	Hum	Ape	Hum	Ape
**Fear**	150.7	63.8	6.24	6.68	162.89	76.92	138.45	50.75	24.17	9.56
**Anger**	136.1	57.7	5.78	6.92	147.39	71.30	124.73	44.18	23.53	8.35
**Happy**	129.8	59.4	6.54	5.69	142.63	70.59	117.00	48.29	19.85	10.45
**Neutral**	126.6	50.3	5.52	6.49	137.41	62.99	115.76	37.57	22.92	7.76
**All.fear**	125.1	52.8	5.33	5.40	135.54	63.37	114.63	42.19	23.46	9.77
**All.anger**	136.0	58.7	5.89	8.52	147.50	75.39	124.41	41.98	23.08	6.89
**All.happy**	136.3	60.1	6.17	6.29	148.36	72.39	124.18	47.74	22.09	9.55
**All.neutral**	145.8	59.8	6.36	6.32	158.28	72.14	133.35	47.30	22.93	9.46

Table S2: Fixation duration estimates at the target emotion compared to all corresponding distractors for human children (Hum) and orangutans (Ape) looking at human facial expressions. Values are derived from general mixed linear model (GLMM) using the function 'summary', predicting estimates, standard errors (Std. Error), upper and lower confidence intervals (upper CI, lower CI), and t-values for the mean fixation duration (in ms) of children (Hum) and orangutans (Ape) on a particular target emotion (fear, anger, happy, neutral) compared to the corresponding all other distractor emotions (All.fear=neutral, happy, anger; All.anger=neutral, happy, fear; All.happy=neutral, fear, anger; All.neutral=fear, anger, happy).

Emotion combination	Estimate		Std. Error		Upper CI		Lower CI		t-value	
	Hum	Ape	Hum	Ape	Hum	Ape	Hum	Ape	Hum	Ape
**Fear**	53780	20977	1934	3054	57569.5	26964.0	49990.2	14990.5	27.8	6.9
**Anger**	48269	18519	1947	2982	52984.2	24363.1	44453.7	12675.3	24.8	6.2
**Happy**	46687	20645	2275	3027	51146.1	26577.4	42227.8	14711.8	20.5	6.8
**Neutral**	45205	16774	1575	2238	48292.1	21161.5	42117.0	12386.6	28.7	7.5
**All.fear**	43846	17348	1695	2174	47168.4	21608.8	40524.0	13087.4	25.9	8.0
**All.anger**	49212	20166	1974	3348	53079.8	26727.9	45343.8	13603.5	24.9	6.0
**All.happy**	48698	20307	1968	2738	52555.1	25673.4	44840.8	14940.7	24.8	7.4
**All.neutral**	52184	19094	2170	3083	56436.9	25136.5	47931.8	13052.1	24.1	6.2

Table S3: Fixation rate estimates at the target emotion compared to a specific distractor emotion for human children (Hum) and orangutans (Ape) looking at human facial expressions. Values are derived from general mixed linear model (GLMM) using the function ‘summary’, predicting estimates, standard errors (Std. Error), upper and lower confidence intervals (upper CI, lower CI), and t-values of the mean fixation rate of children (Hum) and orangutans (Ape) on a particular target emotion (Fear, Anger, Happy, Neutral) compared to another specific distractor emotion (-.fe, -.an, -.ha, -.ne). fe=fear, an=anger, ha=happy, ne=neutral.

Emotion combination	Estimate		Std. Error		Upper CI		Lower CI		t-value	
	Hum	Ape	Hum	Ape	Hum	Ape	Hum	Ape	Hum	Ape
**Anger.fe**	3.04	1.18	0.15	0.21	3.34	1.58	2.74	0.78	19.7	5.7
**Fear.an**	3.56	1.53	0.16	0.27	3.87	2.06	3.25	1.01	22.6	5.7
**Anger.ne**	3.40	1.40	0.17	0.25	3.74	1.88	3.07	0.92	20.2	5.7
**Neutral.an**	3.11	1.14	0.18	0.23	3.46	1.58	2.76	0.70	17.4	5.1
**Fear.ha**	3.50	1.49	0.19	0.20	3.88	1.89	3.12	1.09	18.2	7.4
**Happy.fe**	2.92	1.39	0.18	0.17	3.27	1.73	2.56	1.06	16.0	8.1
**Neutral.ha**	2.96	1.26	0.16	0.22	3.27	1.69	2.64	0.83	18.3	5.7
**Happy.ne**	3.31	1.33	0.18	0.20	3.66	1.72	2.96	0.94	18.4	6.7
**Anger.ha**	3.27	1.54	0.16	0.20	3.59	1.94	2.96	1.15	20.4	7.7
**Happy.an**	3.05	1.52	0.16	0.22	3.37	1.96	2.73	1.08	18.5	6.8
**Fear.ne**	3.70	1.54	0.17	0.17	4.03	1.86	3.38	1.21	22.2	9.3
**Neutral.fe**	2.98	1.20	0.13	0.19	3.22	1.57	2.73	0.83	23.7	6.4

Table S4: Fixation duration estimates at the target emotion compared to a specific distractor emotion for human children (Hum) and orangutans (Ape) looking at human facial expressions. Values are derived from general mixed linear model (GLMM) using the function 'summary', predicting estimates, standard errors (Std. Error), upper and lower confidence intervals (upper CI, lower CI), and t-values of the mean fixation duration (in ms) of children (Hum) and orangutans (Ape) on a particular target emotion (Fear, Anger, Happy, Neutral) compared to another specific distractor emotion (-.fe, -.an, -.ha, -.ne). fe=fear, an=anger, ha=happy, ne=neutral.

**Emotion combination**	**Estimate**		**Std. Error**		**Upper CI**		**Lower CI**		**t-value**	
	Hum	Ape	Hum	Ape	Hum	Ape	Hum	Ape	Hum	Ape
**Anger.fe**	1062.2	353.5	52.5	72.5	1165.0	495.6	959.4	211.3	20.3	4.9
**Fear.an**	1269.1	526.6	56.6	96.4	1380.1	715.6	1158.2	337.6	22.4	5.5
**Anger.ne**	1237.8	443.8	60.6	89.9	1356.6	619.9	1119.1	267.6	20.4	4.9
**Neutral.an**	1108.7	386.7	53.5	80.5	1213.6	544.4	1003.9	229.0	20.7	4.8
**Fear.ha**	1255.7	507.8	62.0	83.8	1377.3	671.9	1134.1	343.6	20.2	6.1
**Happy.fe**	1024.5	491.4	55.3	72.5	1132.9	633.4	916.0	349.4	18.5	6.8
**Neutral.ha**	1075.0	417.2	48.2	76.1	1169.5	566.4	980.5	268.0	22.3	5.5
**Happy.ne**	1173.0	456.1	64.4	91.0	1299.2	634.5	1046.8	277.7	18.2	5.0
**Anger.ha**	1147.7	525.5	56.7	90.1	1258.8	702.1	1036.6	349.0	20.3	5.8
**Happy.an**	1137.3	527.1	64.8	95.3	1264.3	713.8	1010.3	340.4	17.6	5.5
**Fear.ne**	1316.6	464.0	53.5	82.4	1421.4	625.5	1211.8	302.5	24.6	5.6
**Neutral.fe**	1045.2	394.3	45.8	61.5	1134.9	514.8	955.4	273.7	22.8	6.4

Table S5: Fixation rate estimates at the target emotion compared to all distractor emotions for human children (Hum) and orangutans (Ape) looking at orangutan facial expressions. Values are derived from general mixed linear model (GLMM), predicting estimates, standard errors (Std. Error), upper and lower confidence intervals (upper CI, lower CI), and t-values of the mean fixation rate on a particular target. The target reflects one of the emotions SBT (silent bared-teeth display), ROM (relaxed open mouth display), BL (bulging-lip display), and Neutral compared to the corresponding all other distractor expressions (All.sbt=neutral, rom, bl; All.rom=neutral, sbt, bl; All.bl=neutral, sbt, rom; All.neutral=sbt, rom, bl).

Emotion combination	Estimate		Std. Error		Upper CI		Lower CI		t-value	
	Hum	Ape	Hum	Ape	Hum	Ape	Hum	Ape	Hum	Ape
**SBT**	179.3	118.2	9.57	12.65	198.04	142.98	160.51	93.39	18.73	9.34
**ROM**	163.0	108.6	9.01	11.74	180.64	131.56	145.31	85.56	18.08	9.25
**BL**	137.0	113.2	6.68	10.29	150.12	133.32	123.93	93.00	20.51	11.00
**Neutral**	151.9	104.4	8.63	10.94	168.81	125.85	134.99	82.96	17.61	9.54
**All.sbt**	138.4	101.2	6.83	9.77	151.74	120.30	124.96	82.01	20.25	10.36
**All.rom**	157.3	119.0	8.36	11.95	173.64	142.42	140.86	95.58	18.80	9.96
**All.bl**	173.1	111.5	11.22	10.26	195.07	131.64	151.08	91.42	15.42	10.87
**All.neutral**	162.5	112.6	9.57	12.81	181.25	137.73	143.75	87.52	16.98	8.79

Table S6: Fixation duration estimates at the target emotion compared to all corresponding distractor emotions for human children (Hum) and orangutans (Ape) looking at orangutan facial expressions. Values are derived from general mixed linear model (GLMM), predicting estimates, standard errors (Std. Error), upper and lower confidence intervals (upper CI, lower CI), and t-values of the mean fixation duration (in ms) on a particular target. The target reflects one of the emotions SBT (silent bared-teeth display), ROM (relaxed open mouth display), BL (bulging-lip display), and Neutral compared to the corresponding all other distractor expressions (All.Sbt=neutral, rom, bl; All.Rom=neutral, sbt, bl; All.Bl=neutral, sbt, rom; All.neutral=sbt, rom, bl).

Emotion combination	Estimate		Std. Error		Upper CI		Lower CI		t-value	
	Hum	Ape	Hum	Ape	Hum	Ape	Hum	Ape	Hum	Ape
**SBT**	63939	35863	3444	3775	70689.9	43261.8	57188.2	28464.9	18.6	9.5
**ROM**	55148	33799	3089	4466	61203.6	42553.5	49093.3	25045.2	17.9	7.6
**BL**	45864	34128	2600	3843	50960.3	41659.9	40768.0	26595.1	17.6	8.9
**Neutral**	51073	31812	3097	3251	57142.8	38184.0	45003.8	25440.5	16.5	9.8
**All.sbt**	45169	30576	2443	3975	49957.0	38366.0	40381.7	22785.2	18.5	7.7
**All.rom**	53423	35992	2970	3408	59243.7	42671.8	47602.1	29311.7	18.0	10.6
**All.bl**	60488	34982	4143	3669	68608.2	42173.4	52368.2	27789.6	14.6	9.5
**All.neutral**	56944	34054	3362	4201	63533.0	42287.9	50356.0	25819.5	16.9	8.1

Table S7: Fixation rate estimates at the target emotion compared to a specific distractor emotion for human children (Hum) and orangutans (Ape) looking at orangutan expressions. Values are derived from general mixed linear model (GLMM), predicting estimates, standard errors (Std. Error), upper and lower confidence intervals (upper CI, lower CI), and t-values of the mean fixation rate on a particular target emotion (SBT, ROM, BL, NE) compared to another specific distractor emotion (sbt, rom, bl, ne). Note that SBT/sbt = silent bared-teeth, ROM/rom = relaxed open mouth display, BL/bl = bulging-lip display, NE/ne = neutral. Targets are indicates by capital letters, followed by the respective specific distractor abbreviated in lower case letters.

**Emotion combination**	**Estimate**		**Std. Error**		**Upper CI**		**Lower CI**		**t-value**	
	Hum	Ape	Hum	Ape	Hum	Ape	Hum	Ape	Hum	Ape
**SBT.ne**	61.40	37.19	4.09	5.95	69.41	48.85	53.39	25.52	15.0	6.2
**NE.sbt**	44.35	35.41	2.93	3.07	50.09	41.43	38.61	29.39	15.2	11.5
**SBT.bl**	59.58	34.13	3.72	3.93	66.86	41.82	52.29	26.43	16.0	8.7
**BL.sbt**	45.35	31.47	2.50	5.96	50.24	43.15	40.46	19.79	18.2	5.3
**SBT.rom**	58.30	46.88	3.93	5.93	66.01	58.49	50.59	35.26	14.8	7.9
**ROM.sbt**	48.65	34.28	3.17	3.40	54.86	40.94	42.44	27.62	15.4	10.1
**ROM.ne**	56.05	36.44	3.57	5.95	63.04	48.09	49.06	24.78	15.7	6.1
**NE.rom**	52.33	29.44	3.19	4.06	46.07	37.39	58.58	21.48	16.4	7.3
**BL.ne**	45.05	39.00	2.86	3.56	50.65	45.97	39.45	32.03	15.8	11.0
**NE.bl**	55.23	39.56	4.16	4.93	63.38	49.23	47.07	29.89	13.3	8.0
**ROM.bl**	58.28	37.84	4.08	4.61	66.27	46.88	50.28	28.81	14.3	8.2
**BL.rom**	46.63	42.69	2.53	3.14	51.57	48.85	41.68	36.53	18.5	13.6

Table S8: Fixation duration estimates at the target emotion compared to specific distractor emotions for human children (Hum) and orangutans (Ape) looking at orangutan facial expressions. Values are derived from general mixed linear model (GLMM), predicting estimates, standard errors (Std. Error), upper and lower confidence intervals (upper CI, lower CI), and t-values of the mean fixation duration (in ms) on a particular target emotion (SBT, ROM, BL, NE) compared to another specific distractor emotion (sbt, rom, bl, ne). SBT/sbt = silent bared-teeth, ROM/rom = relaxed open mouth display, BL/bl = bulging-lip display, NE/ne = neutral. Targets are indicates by capital letters, followed by the respective specific distractor abbreviated in lower case letters.

Emotion combination	Estimate		Std. Error		Upper CI		Lower CI		t-value	
	Hum	Ape	Hum	Ape	Hum	Ape	Hum	Ape	Hum	Ape
**SBT.ne**	22316.3	10867.6	1370.8	1802.2	25003.0	14399.9	19629.6	7335.3	16.3	6.0
**NE.sbt**	14414.5	10449.0	1077.0	1086.3	16525.4	12578.1	12303.7	8319.9	13.4	9.6
**SBT.bl**	21471.3	10423.9	1485.7	1334.8	24383.3	13040.2	18559.4	7807.7	14.5	7.8
**BL.sbt**	14520.5	9687.3	862.5	2330.3	16211.1	14254.7	12830.0	5120.0	16.8	4.2
**SBT.rom**	20151.4	14571.8	1375.4	1846.7	22847.1	18191.3	17455.7	10952.3	14.7	7.9
**ROM.sbt**	16234.3	10439.3	1058.1	1160.7	18308.2	12714.3	14160.4	8164.3	15.3	9.0
**ROM.ne**	19022.5	11631.3	1262.9	2106.9	21497.8	15760.9	16547.2	7501.8	15.1	5.5
**NE.rom**	17533.5	8534.4	1222.3	970.5	19929.2	10436.6	15137.9	6632.3	14.4	8.8
**BL.ne**	15605.7	11554.7	1130.7	1058.1	17821.8	13628.4	13389.6	9480.9	13.8	10.9
**NE.bl**	19125.2	12828.8	1427.8	1680.4	21923.6	16122.4	16326.8	9535.3	13.4	7.6
**ROM.bl**	19891.7	11728.7	1464.2	1809.1	22761.4	15274.4	17021.9	8183.0	13.6	6.5
**BL.rom**	15738.0	12885.5	949.2	956.2	17598.4	14759.7	13877.5	11011.3	16.6	13.5

Table S9: Fixation duration and fixation rate comparisons on same vs. other species stimuli. Values are derived from a paired t-test, comparing children′s fixation duration and fixation rate on orangutan stimuli (CsA) and human stimuli (CsH) as well as orangutans′ fixations on orangutan stimuli (AsA) and human stimuli (AsH).

**Paired t-test**									
		**M**	**SD**	**Standard error**	**Lower CI**	**Upper CI**	**T**	**df**	***p***
Fixation rate	CsA vs. CsH	0,003	0,039	0,011	-0,022	0,028	0,293	11	0,775
	AsA vs. AsH	0,077	0,026	0,008	0,060	0,093	10,06	11	0
Fixation duration	CsA vs. CsH	-2,435	16,855	4,866	-13,144	8,274	-0,5	11	0,627
	AsA vs. AsH	20,703	9,242	2,668	14,831	26,574	7,76	11	0
